# Lungs From Donors ≥70 Years of Age for Transplantation—Do Long-Term Outcomes Justify Their Use?

**DOI:** 10.3389/ti.2023.11071

**Published:** 2023-04-13

**Authors:** Wiebke Sommer, Maximilian Franz, Khalil Aburahma, Akylbek Saipbaev, Katharina Flöthmann, Pavel Yablonski, Murat Avsar, Igor Tudorache, Mark Greer, Axel Haverich, Tobias Welte, Christian Kuehn, Jawad Salman, Gregor Warnecke, Fabio Ius

**Affiliations:** ^1^ Department of Cardiac Surgery, University of Heidelberg, Heidelberg, Germany; ^2^ German Center for Lung Research, Deutsches Zentrum Lungenforschung (DZL), BREATH, Hannover, Germany; ^3^ Department of Cardiothoracic, Vascular and Transplantation Surgery, Hannover Medical School, Hannover, Germany; ^4^ Department of Cardiac Surgery, University of Duesseldorf, Duesseldorf, Germany; ^5^ Department of Pulmonology, Hannover Medical School, Hannover, Germany

**Keywords:** lung transplantation, extended criteria donor lungs, marginal donor lungs, old donor lungs, lung donor characteristics

## Abstract

Donor shortages have led transplant centers to extend their criteria for lung donors. Accepting lung donors ≥70 years of age has previously shown good short-term outcomes; however, no mid- and long-term outcome data on these extended criteria donors has been published to date. In this study, all patients who underwent lung transplantation between 06/2010 and 12/2019 were included in the analysis, and the outcomes were compared between patients receiving organs from donors <70 years of age and patients transplanted with lungs from donors ≥70 years of age. Among the 1,168 lung-transplanted patients, 62 patients received lungs from donors ≥70 years of age. The recipient age of those receiving older organs was significantly higher, and they were more likely to suffer from obstructive lung disease. Older donors were exposed to significantly shorter periods of mechanical ventilation prior to donation, had higher Horowitz indices, and were less likely to have smoked. The postoperative time on mechanical ventilation, time on ICU, and total hospital stay were comparable. The overall survival as well as CLAD-free survival showed no differences between both groups in the follow-up period. Utilization of lungs from donors ≥70 years of age leads to excellent mid- and long-term results that are similar to organs from younger donors when the organs from older donors are carefully preselected.

## Graphical Abstract

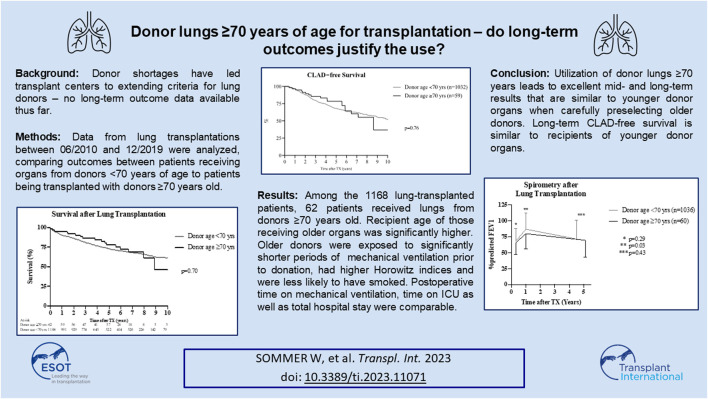

## Introduction

Given the known global shortage of ideal suitable donor organs for lung transplantation, obtaining more organs from the existing donor pool has been one tool used to optimize patient care in end-stage lung disease. As a result, utilization of non-ideal donor lungs from “extended-criteria” donors has become clinical routine in large lung transplant programs (1–4).

The lung donor age has steadily increased in Europe over the past number of years, with a reported median donor age of 51 years in 2018. In contrast, the median lung donor age in North America remains much lower at approximately 33 years for the past decade (5). Given these substantial geographic differences, countries with older organ donors are confronted with extended criteria organ offers on a daily basis in order to provide optimized patient care.

The impact of donor age on lung transplant outcomes and the clinical feasibility have been reviewed by multiple transplant centers in the past, with conflicting conclusions. More recent analyses have shown that an advanced donor age of >55 years does not appear to have a negative impact on recipient survival, especially in older recipients (6–9), whereas earlier analyses tended to show survival disadvantages in candidates receiving lungs from older donors (10, 11).

We have previously described outcomes using donor lungs from donors ≥70 years, finding no early survival disadvantage for up to 3 years after transplantation (7). Spirometry results in this early analysis indicated better results for recipients with an obstructive underlying disease pattern prior to transplantation, as compared to restrictive pulmonary disease.

However, longer-term follow-up of these “extended-criteria” donor organs has not yet been reported. The aim of this study is, therefore, to summarize the long-term follow-up of recipients of donor lungs from donors aged 70 and older in comparison to recipients of donor organs from donors younger than 70 years of age.

## Patients and Methods

### Patient Groups

All patients who underwent lung transplantation between 06/2010 and 12/2019 at Hannover Medical School were included in the retrospective analysis. Lung recipients were divided in two groups: patients transplanted with lungs from donors <70 years and patients transplanted with lungs from donors ≥70 years. Outcome parameters, including pre-, peri-, and postoperative clinical parameters, as well as recipient overall survival and freedom from chronic lung allograft dysfunction (CLAD) were recorded and compared between the two groups.

All patients provided written informed consent for data utilization for scientific purposes at the time of listing for transplantation.

### Variable Definition

The primary composite outcome, graft survival, was defined as patient and graft survival and included patient mortality and the need for retransplantation. Primary graft dysfunction (PGD) was defined according to current International Society for Heart Lung Transplantation (ISHLT) guidelines (12).

Graft function was evaluated at regular outpatient visits and included surveillance biopsies as well as home spirometry testing. Predicted FEV1 was calculated for each recipient utilizing the formula FEV1 = race*((0.0395*height)−(0.025*age)−2.6). Since all recipients are Caucasian in the analyzed cohorts, ‘race’ was substituted by “1” in the formula. The measured FEV1 was then expressed as the %predicted FEV1.

CLAD was defined following current ISHLT guidelines as a persistent decline of FEV1 ≥20% from baseline in the absence of other conditions causing pulmonary impairment (13).

### Donor Management

All donor organs were offered to our center by Eurotransplant. Within the regular LAS-based allocation process, organs were allocated for a specifc recipient, whereas organs in the rescue allocation process were accepted by the center and the recipient was chosen by the transplant center. Organ assessment and preservation were the same for lungs of donors <70 and of donors ≥70 years of age. Following endobronchial as well as macroscopic assessment of the donor lung during procurement, the donor organ was accepted by a surgical team from our center. Organs with irreversible macroscopic signs of parenchymal alterations such as emphysema were not accepted.

### Recipient Management

Recipient management at our institution has been previously reported and did not differ between groups (14). All recipient characteristics were recorded as previously reported and spirometry results were included after discharge following the initial hospital stay, 1 year after transplantation, and during the last follow-up visit at the outpatient clinic. Calculation of recipient-specific %predicted FEV1 was performed as previously reported (7). The clinical routine in our program includes, if hemodynamically necessary, intraoperative extracorporeal support using veno-arterial extracorporeal membrane oxygenation (ECMO) instead of conventional cardiopulmonary bypass (CPB). CPB is only used if additional cardiac surgery is performed, which is technically not feasible with ECMO (e.g., atrial septal defect closure). It should be noted that, as per our centre’s protocol, recipients with an underlying diagnosis of primary pulmonary hypertension received postoperative prolonged veno-arterial ECMO treatment for left ventricular remodeling as a planned treatment strategy (15).

### Statistics

Retrospective analysis of all parameters was performed using GraphPad Prism, Version 8.0 (San Diego, Ca, USA). Multivariate analysis was performed using SPSS 28.0.1.1 (IBM, Armonk, NY, USA).Variables are summarized as percentages, mean ± standard deviation (SD), or median (interquartile range, IQR). A Mann–Whitney *U* test was performed to test differences between continuous variables. Outcome-free survivals were calculated using the Kaplan-Meier method and were compared by using a log-rank test. *p* values < 0.05 were considered statistically significant.

## Results

### Patient Groups

A total of 1,168 patients underwent lung transplantation at Hannover Medical School between 06/2010 and 12/2019, of which 62 (5.3%) recipients received allografts from donors ≥70 years of age and the remaining 1,106 (94.7%) patients allografts from donors <70 years of age. The median follow-up was 8.9 years.

### Recipient Characteristics

Patients who received lungs from donors ≥70 years of age were significantly older compared to recipients of organs from donors <70 years of age (median (IQR) 57 (54; 62) vs. 51 (36; 58) years of age; *p* < 0.0001). The body mass index of recipients who received organs from older donors was slightly higher than recipients of organs from younger donors (Table 1).

**TABLE 1:** Recipient preoperative characteristics.

	Donor <70 years of age (*n* = 1,106)	Donor ≥70 years of age (*n* = 62)	*p*-value
Age (median; IQR)	51 (36; 58)	57 (54; 62)	<0.0001
Female (%)	48.2	53.2	0.51
BMI (mean ± SD)	22.1 ± 4.3	23.1 ± 3.6	0.04
Underlying Disease (n; %)			
Emphysema	305; 27.6	25; 40.3	0.04
Fibrosis	350; 31.6	25; 40.3	0.16
Cystic fibrosis	231; 20.9	3; 4.8	0.003
Primary pulmonary hypertension	68; 6.1	3; 4.8	0.79
Re-transplant for CLAD	74; 6.7	-	0.05
Sarcoidosis	37; 3.3	4; 6.5	0.27
Other	41; 3.7	2; 3.2	0.84
Lung allocation score (median; IQR)	36 (33; 42.5)	34.9 (32.5; 39.3)	0.18
Time on waiting list (days) (mean ± SD)	220.2 ± 454.7	175.4 ± 296.1	0.56
Pulmonary artery pressure (mean ± SD)	27.3 ± 14.2	27.6 ± 12.8	0.42
Preop mechanical ventilation (n; %)	36; 3.3	2; 3.2	>0.99
Preop intensive care unit (n; %)	113; 10.2	5; 8.1	0.67
Preop ECMO (n; %)	5; 8.1	73; 6.6	0.79

BMI, body mass index; CLAD, chronic lung allograft dysfunction; ECMO, extracorporeal membrane oxygenation.

The distribution of transplant indications differed significantly between both groups. Organs from older donors were more likely to be offered to candidates suffering from chronic obstructive pulmonary disease (COPD) (40.3% vs. 27.6%, *p* = 0.04). In contrast, candidates with cystic fibrosis were more often transplanted with organs from younger donors (20.9% vs. 4.8%; *p* = 0.003). Lung retransplantation for CLAD was performed solely with organs from donors aged <70 years (*p* = 0.05) (Table 1).

The median lung allocation score (LAS; *p* = 0.18) and time on the waiting list (*p* = 0.56) showed no significant difference between groups.

Regarding the preoperative risk profile, no differences in the need for preoperative mechanical ventilation (3.2% vs. 3.3%; *p* < 0.99), preoperative ICU treatment (8.1% vs. 10.2%; *p* = 0.67), or preoperative ECMO (6.6% vs. 8.1%; *p* = 0.79) were observed (Table 1).

### Donor Characteristics

The median donor age in the ≥70 years of age group was 73 years of age (71; 75) vs. 47 years of age (34; 56) in the <70 years of age group, with a similar gender distribution between both groups (*p* = 0.19). Older donors had significantly shorter exposure to mechanical ventilation prior to procurement (3 (2; 4) vs. 4 (2; 7) days; *p* = 0.0007) but showed a higher Eurotransplant donor score compared to younger organ donors (8.7 ± 1.1 vs. 7.9 ± 1.6; *p* < 0.0001) (16). The oxygenation capacity (PaO_2_ at 100% FiO_2_, mmHg) of donors aged ≥70 years was higher compared to donors <70 years of age (412.5 (356; −469.5) vs. 384.0 (316; −448); *p* = 0.01). Additionally, older donors were less likely to have a smoking history compared to younger organ donors (12.9% vs. 42.1%; *p* < 0.0001). No organ donors aged ≥70 years of age showed signs of pulmonary contusion or aspiration (Table 2).

**TABLE 2:** Donor characteristics.

	Donor <70 years of age (*n* = 1,106)	Donor ≥70 years of age (*n* = 62)	*p*-value
Age (years) (median; IQR)	47 (34; 56)	73 (71; 75)	<0.0001
Female (n; %)	559; 50.5	37; 59.7	0.19
BMI (mean ± SD)	25.7 ± 5.0	26.2 ± 2.9	0.10
Time on mechanical ventilation (days) (median; IQR)	4 (2; 7)	3 (2; 4)	0.0007
ET donor score (mean ± SD)	7.9 ± 1.6	8.7 ± 1.1	<0.0001
PaO_2_ (FiO_2_ 1.0) (median; IQR)	384.0 (316; 448)	412.5 (356; 469.5)	0.01
History of smoking (n; %)	465; 42.1	8; 12.9	<0.0001
Contusion (n; %)	106; 9.6	-	0.009
Aspiration (n; %)	70; 6.3	-	0.04
Use of *ex vivo* lung perfusion (n; %)	65; 5.9	4; 6.5	0.85

BMI, body mass index; ET donor score: Eurotransplant donor score.

### Intraoperative Characteristics

The majority of lung transplantations were performed as bilateral minimally-invasive surgeries, with no differences between groups. The need for extracorporeal support did not differ between groups (32.2% vs. 26.6% *p* = 0.38). Notably, the majority of patients requiring extracorporeal support intraoperatively were put on veno-arterial ECMO. Cardiopulmonary bypass was only used in a minority of cases, in which additional cardiac surgery was performed (2.1% vs. 1.6%; *p* = 0.80). The cold ischemic times of the first (*p* = 0.29) and second implanted lung (*p* = 0.91) did not differ between groups (Table 3).

**TABLE 3:** Recipient intra- and postoperative characteristics.

	Donor <70 years of age (*n* = 1,106)	Donor ≥70 years of age (*n* = 62)	*p*-value
Minimally-invasive (n; %)	1,034; 93.5	56; 90.3	0.43
Bilateral lung transplantation (n; %)	1,075; 97.2	61; 98.4	0.72
Intraoperative use of cardiopulmonary bypass (n; %)	23; 2.1	1; 1.6	0.80
Intraoperative use of ECMO (n; %)	294; 26.6	20; 32.3	0.38
Ischemic time; first side (min) (mean ± SD)	414 ± 122.1	396.3 ± 122.9	0.29
Ischemic time; second side (min) (mean ± SD)	527.9 ± 129.5	526.6 ± 135.7	0.91
ECMO postoperative (n; %)	104; 9.4	6; 9.7	0.94
ECMO postoperative per protocol^a^ (n; %)	84; 7.6	4; 6.5	0.81
PGD score @24h (mean ± SD)	0.51 ± 0.91	0.53 ± 0.95	0.99
PGD score @48h (mean ± SD)	0.51 ± 0.90	0.55 ± 0.92	0.60
PGD score @72h (mean ± SD)	0.46 ± 0.85	0.50 ± 0.94	0.94
PGD 2 or 3 @72h (n; %)	146; 13.2	9; 14.5	0.84
Postoperative new dialysis (n; %)	99; 8.9	5; 8.1	0.83
Dialysis at discharge (n; %)	56; 5.1	2; 3.2	0.58
Mechanical ventilation postop (days) (median; IQR)	1 (1; 1)	1 (1; 1)	0.68
ICU stay (days) (median; IQR)	2 (1; 5)	2 (1; 4.5)	0.65
Total hospital stay (days) (median; IQR)	23 (21; 31)	23 (21; 32.5)	0.58
1-year survival (%)	90.2	95.1	0.21
3-year survival (%)	80.9	86.4	0.28
5-year survival (%)	73.2	77.8	0.34

^a^ Centre’s protocol for postoperative ECMO in pulmonary arterial hypertension.

ECMO, extracorporeal membrane oxygenation; PGD, primary graft dysfunction; ISHLT PGD score; ICU, intensive care unit.

### Postoperative Characteristics

The rates of postoperative ECMO were similar in both cohorts (9.7% vs. 9.4%; *p* = 0.94). The majority of these ECMO treatments resulted from our centre’s protocol for postoperative remodeling of the left ventricle in patients with severe pulmonary arterial hypertension (6.5% vs. 7.6%; *p* = 0.81) (15).

The primary graft dysfunction (PGD) score grade 3 at 24 h (*p* = 0.99), 48 h (*p* = 0.60) and 72 h (*p* = 0.94) after transplantation did not differ between groups.

Postoperative characteristics, including mechanical ventilation (*p* = 0.68), intensive care stay (*p* = 0.65), and total hospital stay times (*p* = 0.58), did not differ between groups (Table 3).

### Survival

**FIGURE 1:**
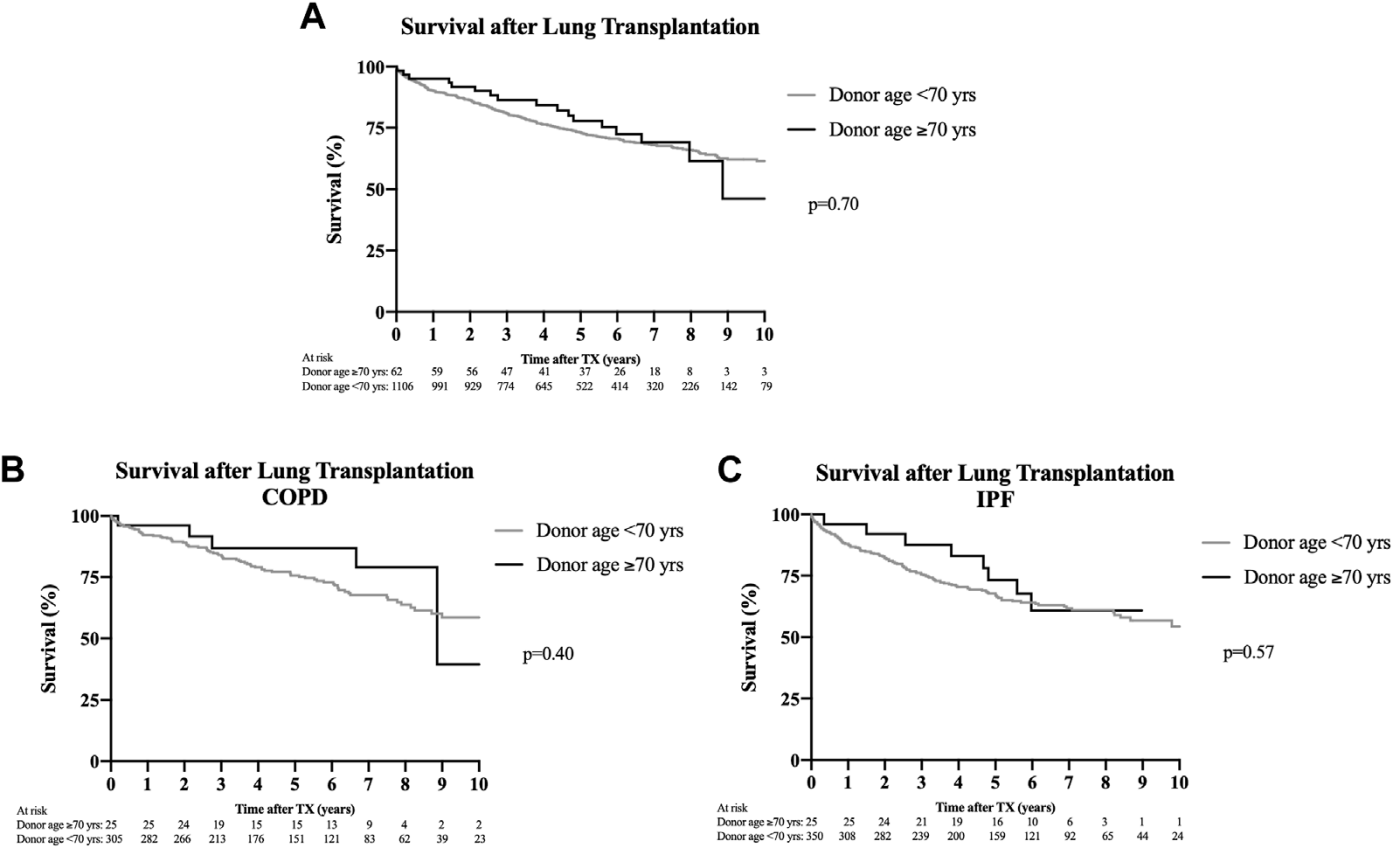
Survival after lung transplantation. Kaplan-Meier analyses. **(A)** Overall survival between recipients who received organs from donors aged ≥70 years and recipients of organs from donors aged <70 years, showing no significant difference (*p* = 0.70) up to 10 years after lung transplantation. **(B)** Stratification of posttransplant survival in patients with COPD as transplant indication differentiating according to donors aged ≥70 and <70 years. No significant difference in survival up to 10 years after transplantation was detectable (*p* = 0.40). **(C)** Stratification of posttransplant survival in patients with idiopathic pulmonary fibrosis according to donors aged ≥70 or <70 years. No survival difference up to 10 years after transplantation was noticeable (*p* = 0.57).

No differences in overall survival were observed between cohorts (*p* = 0.71) (Figure 1A), as measured at 1, 3, and 5 years (*p* = 0.21; *p* = 0.28; and *p* = 0.34) (Table 3). Patients who received lungs from donors aged ≥70 years showed no survival difference with respect to their underlying disease as compared to recipients of organs from younger donors in the same disease cohort (Figures 1B, C).

### Chronic Lung Allograft Dysfunction

**FIGURE 2:**
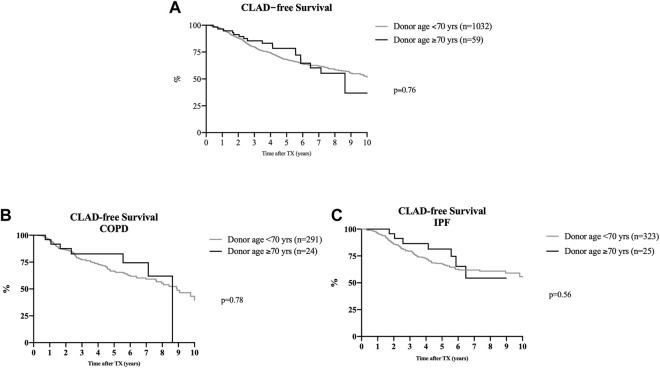
CLAD-free survival after lung transplantation. Kaplan-Meier analyses. **(A)** Overall CLAD-free survival following lung transplantation utilizing organs from donors aged ≥70 years or >70 years, showing no difference between both groups (*p* = 0.76) within the first 10 years after transplantation. **(B)**: Stratification of CLAD-free survival in patients with COPD who received a lung transplantation from donors either ≥70 or <70 years of age. Donor age had no impact on the development of CLAD (*p* = 0.78). **(C)** Stratification of CLAD-free survival in patients with idiopathic pulmonary fibrosis who underwent lung transplantation with organs from donors aged ≥70 or <70 years of age. The incidence of CLAD was similar in both groups (*p* = 0.56).

The incidence of CLAD did not differ between groups (Figure 2A). CLAD-free survival in recipients of organs from donors ≥70 years of age as compared to recipients of organs from donors <70 years after 3 and 5 years were 85.5% vs. 79.7% and 78.5% vs. 68.1%, respectively. Stratification of graft survival in patients transplanted for COPD and pulmonary fibrosis according to donor ages of <70 or >70 years did not differ between groups (Figures 2B, C).

### Postoperative Spirometry Results

**FIGURE 3:**
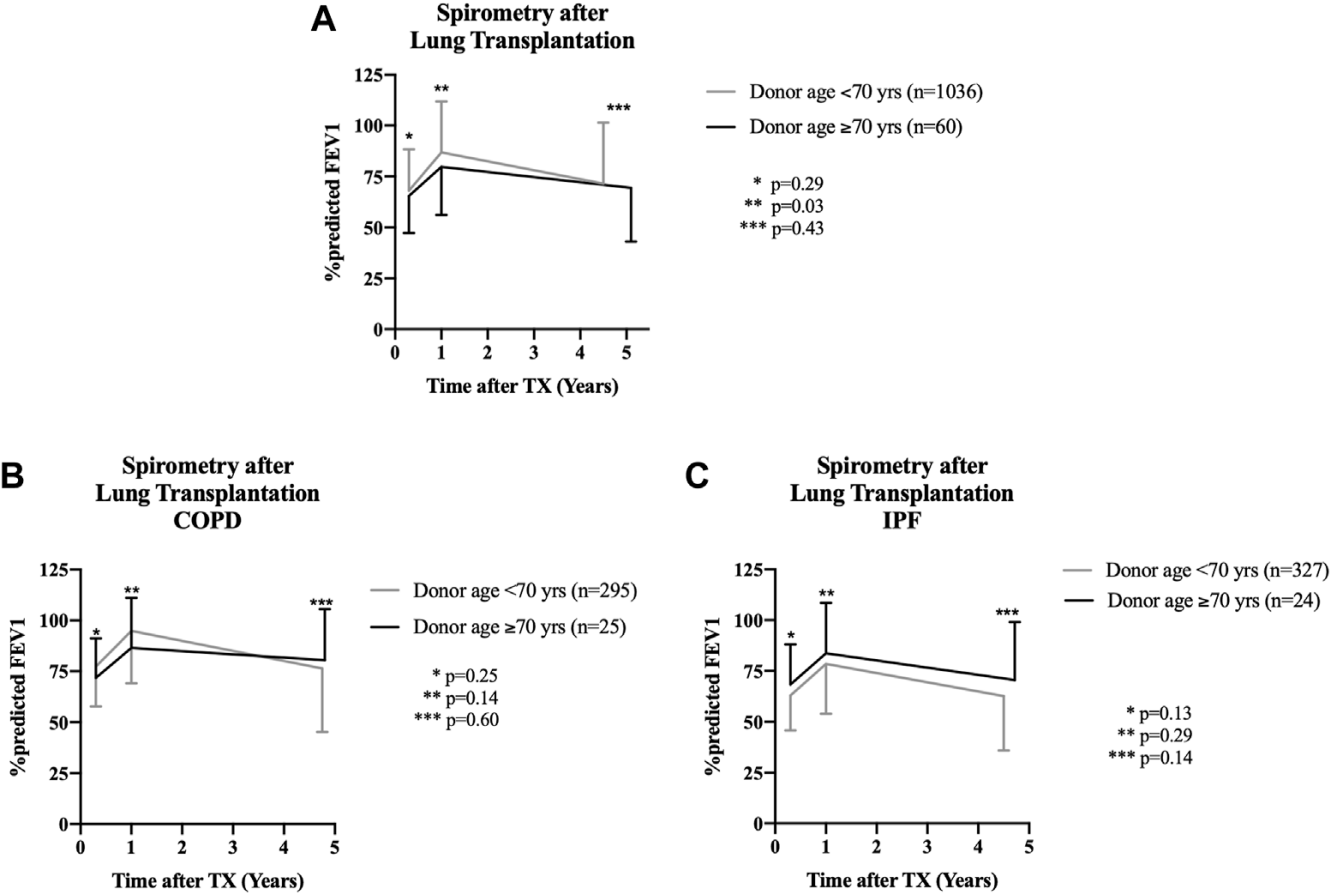
Spirometry results for recipients after lung transplantation. Values are shown as mean ± standard deviation of the %predicted FEV1 at teh time of discharge from initial hospital stay (1st value), at 1 year after transplantation (2nd value), and at last follow-up in the outpatient clinic (3rd value). **(A)** Comparison of recipients of organs from donors aged ≥70 years with outcomes of patients who received lungs from donors aged <70 years. No functional spirometry difference was found at the time of discharge from the hospital (*p* = 0.29), but recipients of organs from donors <70 years of age showed a statistically significant better %predicted FEV1 at 1 year following transplantation (*p* = 0.03). This difference was no longer detectable at last follow-up after a median of 4.5 years (<70 years of age cohort) and 5.1 years (≥70 years of age cohort) (*p* = 0.43). **(B)** Sub-analysis of patients with the underlying disease COPD. No functional differences in spirometry results was detectable throughout the entire follow-up period when comparing donors aged ≥70 years and <70 years of age. **(C)**: Sub-analysis of patients with idiopathic pulmonary fibrosis undergoing lung transplantation with organs from donors aged ≥70 or <70 years of age. No difference in spirometry results was detectable within the first 5 years after transplantation for both cohorts.

FEV1 (%predicted) did not differ between groups at discharge (63.2% (52.2; 78.4) vs. 66.4% (55; 80.5); *p* = 0.29) (Figure 3A). One year after lung transplantation, recipients of organs from donors <70 years of age showed a significantly higher FEV1 (%predicted) as compared to recipients of lungs from donors aged ≥70 years (76.8% (63; 93.2) vs. 86.0% (70; 104); *p* = 0.03). This significant difference between both cohorts diminished in the following years after lung transplantation, showing similar %predicted FEV1 values at the last outpatient follow-up visit (70.5% (53; 87.3) vs. 73.3% (50; 94); *p* = 0.43) (Figure 3A). Stratification of FEV1 in patients with COPD and pulmonary fibrosis according to donor ages of <70 or ≥70 years did not show any difference between groups (Figures 3B, C).

### Donor Age Is Not a Risk Factor for Mortality or CLAD Development

In multivariable Cox regression analysis, which included multiple recipient- and donor-specific variables as well as procedure intraoperative variables (Table 4), donor age was not a risk factor for recipient mortality (*p* = 0.50) or the development of CLAD (*p* = 0.67) (Table 5).

**TABLE 4:** Variables included in multivariable Cox Regression Analysis.

Variables
Donor age ≥70 years
Recipient data
Age
Female sex
BMI recipient
Emphysema
Fibrosis
Cystic fibrosis
Primary pulmonary hypertension
Re-transplant for CLAD
Sarcoidosis
Other
Lung allocation sore
Time on waiting list
Pulmonary artery pressure
Preoperative mechanical ventilation
Preoperative Intensive Care Unit
Preoperative ECMO
Donor data
Female sex
BMI
Time on mechanical ventilation
PaO_2_ (FiO_2_ = 1.0)
History of smoking
Contusion
Aspiration
Intraoperative data
Minimal invasive access
Cardiopulmonary bypass
ECMO
Ischemic time first side
Ischemic time second side

CLAD, chronic lung allograft dysfunction; ECMO, extracorporeal membrane oxygenation; FiO_2_, Fraction of inspired oxygen; BMI, body-mass index.

**TABLE 5:** Multivariable cox regression analysis.

Variable	Multivariable		
Mortality (*n* = 341)	HR	95% CI	*p*-value
Donor age ≥70 years	0.826	0.475–1.438	0.50
Recipient age	1.014	1.004–1.025	0.008
Intraoperative ECMO	1.706	1.286–2.264	<0.001
First lung ischemic time	1.002	1.000–1.003	0.006
CLAD Incidence (*n* = 352)			
Donor age ≥70 years	1.130	0.65–1.964	0.67
History of smoking	1.527	1.180–1.977	0.001

ECMO, extracorporeal membrane oxygenation; CLAD, chronic lung allograft dysfunction; CI, confidence interval; HR, hazard ratio.

Risk factors associated with recipient mortality included recipient age (*p* = 0.008), intraoperative utilization of ECMO (*p* < 0.001), and ischemic time of the first lung (*p* = 0.006). A donor history of smoking was identified as a risk factor for the diagnosis of CLAD (*p* = 0.001) (Table 5).

## Discussion

Over the past two decades, discordance between the consistently high number of candidates awaiting lung transplantation and the number of available donor organs has led experienced transplant centers toward accepting “extended-criteria” donor organs in order to reduce waiting list mortality (3, 4). Questions remain however, regarding the limits of acceptability, as to what degree “extended-criteria” donor lungs can be used for transplantation without compromising recipient outcomes. Retrospective analyses have already demonstrated no adverse outcomes when using donor lungs with acute pulmonary embolism (17, 18) impaired oxygenation (19, 20), or contusion (21). Regarding donor age, multiple analyses have shown good results for lungs from donors >55 years of age (22, 23) however, the upper donor age limit in lung transplantation remains under discussion.

As per our program policy, donor offers are not declined solely because of advanced donor age, but such offers were targeted toward older recipients where possible in the allocation process. Organs from older donors, with additional risk factors such as a relevant history of smoking, severe infiltrates, contusion, or parenchymal alterations, were usually rejected outright upon offer or by an experienced surgeon at procurement. Since the majority of lungs from donors aged ≥70 years were accepted in the rescue allocation process, recipient selection for these organs was performed by our transplant center. Careful recipient selection was also undertaken with regards to anticipated intra- and postoperative risks and retransplantation as well as younger candidates were excluded. Through this combination of donor and recipient selection, utilization of organs from donors aged ≥70 years has facilitated meaningful mid- and long-term outcomes that were comparable to those seen in recipients of organs from younger donors. Both cohorts demonstrated statistically insignificant 1-, 3-, and 5-year survival differences, with recipients of organs from donors aged <70 years showing non-inferior survival rates (1-year: 95.1% vs. 90.2%; 3-year: 86.4% vs. 80.9%; 5-year: 77.8% vs. 73.1%), which for all time points lie above ISHLT reported averages (24).

These findings are in contrast to existing analyses of the UNOS database, which identified a 2.14 fold increased risk in 1-year mortality in recipients of lungs from donors aged ≥65 years (25). This report however, did not include information on “recipient-related” risks that may have contributed to impaired early survival. We would argue that this again underlines the importance of cautious recipient selection for lungs from older donors. Another important aspect in managing all forms of “extended-criteria” donor organs may well be center volume and the inherent level of experience with marginal donor organs as well as recipient matching. Registry analyses usually comprise both entities and do not differentiate results between large- and low-volume centers. Given the previously reported negative impact of low center volume on lung transplant outcome, these results may well be further aggravated in the field of “extended-criteria” donor organs (26, 27).

The physiological differences in the characteristics of advanced age lungs that may influence outcomes, either negatively or indeed positively after transplantation, remain unknown. By selecting organs with no or little smoking history and with careful visual inspection of parenchymal alterations such as bullae or rarefication, moderate or severe age-related obstructive pulmonary disease may be excluded. Temporary disconnection of the ventilator when inspecting the organ in the donor should be advocated, to assess the capacity of the organ to collapse as an important indicator of possible airway obstruction. Similarly, an elevated precapillary pulmonary artery pressure can be quickly excluded invasively within the procurement setting. Applying these measures routinely during the acceptance process of lungs from older donors may assist in achieving similar functional outcomes, with both cohorts showing comparable spirometry results during long-term follow-up. It should be noted that we previously found lower spirometry results in the first postoperative year in patients with pulmonary fibrosis who received organs from donors ≥70 years of age as compared to recipients with an obstructive underlying pulmonary disease pattern (7). This finding was not detectable in longer follow-up data in this larger cohort, showing comparable %predicted FEV1 courses in the individual disease cohorts. Most likely, increased patient numbers led to these results.

Although of critical importance, graft function is however only one consideration. Concerns continue to be expressed regarding the utilization of advanced age donor lungs and the potentially higher risk of transferring malignant tumors to recipients. While understandable, little corroborating data supporting this argument exists. The underlying concerns are not entirely organ-specific, and would be considered similarly legitimate in abdominal organ transplantation, where older donors have been used regularly for decades. Despite this, donor-derived malignant tumor transmission remains an extremely rare event in solid organ transplantation (28–30). Age does appear to increase risk, and, as a consequence, additional measures such as routine computer tomography imaging of potential donors ≥65 years of age prior to organ donation may attenuate the risk of utilizing organs with cancer suspicious structures.

Regarding candidate considerations, lung transplantation in selected older recipients have been performed in high volume transplant centers with acceptable outcomes. However, most received lungs from donors aged <40 years (31). Analogous to the Eurotransplant senior program for kidney transplantation established in 1999 (32), an ‘advanced age’ focused donor-recipient matching program for lung transplantation could potentially assist in providing adequate patient outcomes whilst fully utilizing the existing donor pool. Given that donor lung utilization in donors aged ≥65 years remains <3% in the United States and low within Eurotransplant associated countries (33), such a program may benefit older patients with obstructive pulmonary disease pattern, who usually have minimal perioperative risk factors but also low lung allocation scores and limited probability of receiving a timely transplantation in the regular allocation process. Moreover, senior recipients show no survival impairment when receiving lungs from donors aged ≥60, making this approach clinically relevant (34, 35). This finding is in line with our findings, which show that donor age is not a risk factor for recipient mortality or the development of CLAD. This is especially important, since enrolment in such age-restricted programs requires informed consent of the candidate.

## Limitations

The dataset comprises the known limitations of a single-center retrospective analysis. The overall number of analyzed transplantations using donors aged ≥70 years remains low as compared to larger registry analyses; however, in contrast to those, more detailed follow-up information, including spirometry results as well CLAD incidence, were available.

## Conclusion

In conclusion, the utilization of lungs from donors ≥70 years of age presents a feasible option, especially for advanced age recipients, facilitating comparable early-, mid-, and long-term outcomes regarding survival, CLAD development, and spirometry as compared to transplantations utilizing organs from donors younger than 70 years of age. These results can be achieved by carefully selecting both suitable donors as well as recipients.

## Data Availability

The raw data supporting the conclusion of this article will be made available by the authors, without undue reservation.

## Abbreviations

BMI, body mass index; CLAD, chronic lung allograft dysfunction; COPD, chronic obstructive pulmonary disease; ECMO, extracorporeal membrane oxygenation; ET, Eurotransplant; FEV1, forced expiratory pressure in 1 second; LAS, lung allocation score; ICU, intensive care unit; ISHLT, International Society for Heart and Lung Transplantation; LAS, lung allocation score; PGD, primary graft dysfunction; UNOS, united network for organ sharing.

## References

[1] Snell GI Paraskeva M Westall GP. Donor Selection and Management. Semin Respir Crit Care Med (2013) 34(3):361–70. 10.1055/s-0033-1348464 23821510

[2] Wadowski B Chang SH Carillo J Angel L Kon ZN. Assessing Donor Organ Quality According to Recipient Characteristics in Lung Transplantation. J Thorac Cardiovasc Surg (2022) 65(2):532–43. 10.1016/j.jtcvs.2022.03.014 35461708

[3] Christie IG Chan EG Ryan JP Harano T Morrell M Luketich JD National Trends in Extended Criteria Donor Utilization and Outcomes for Lung Transplantation. Ann Thorac Surg (2021) 111(2):421–6. 10.1016/j.athoracsur.2020.05.087 32663473

[4] Sommer W Kühn C Tudorache I Avsar M Gottlieb J Boethig D Extended Criteria Donor Lungs and Clinical Outcome: Results of an Alternative Allocation Algorithm. J Heart Lung Transpl (2013) 32(11):1065–72. 10.1016/j.healun.2013.06.021 23953918

[5] Chambers DC Zuckermann A Cherikh WS Harhay MO Hayes D Jr Hsich E The International Thoracic Organ Transplant Registry of the International Society for Heart and Lung Transplantation: 37th Adult Lung Transplantation Report - 2020; Focus on Deceased Donor Characteristics. J Heart Lung Transpl (2020) 39(10):1016–27. 10.1016/j.healun.2020.07.009 PMC7737221 32782073

[6] Hayes D Jr Black SM Tobias JD Higgins RS Whitson BA. Influence of Donor and Recipient Age in Lung Transplantation. J Heart Lung Transpl (2015) 34(1):43–9. 10.1016/j.healun.2014.08.017 25301358

[7] Sommer W Ius F Salman J Avsar M Tudorache I Kuhn C Survival and Spirometry Outcomes after Lung Transplantation from Donors Aged 70 Years and Older. J Heart Lung Transpl (2015) 34(10):1325–33. 10.1016/j.healun.2015.06.002 26186805

[8] Hecker M Hecker A Kramm T Askevold I Kuhnert S Reichert M Use of Very Old Donors for Lung Transplantation: a Dual-centre Retrospective Analysis. Eur J Cardiothorac Surg (2017) 52(6):1049–54. 10.1093/ejcts/ezx202 28977370

[9] Renard R Girault A Avramenko-Bouvier A Roussel A Cerceau P Pellenc Q Outcome of Lung Transplantation Using Grafts from Donors over 65 Years of Age. Ann Thorac Surg (2021) 112(4):1142–9. 10.1016/j.athoracsur.2020.10.018 33171173

[10] De Perrot M Waddell TK Shargall Y Pierre AF Fadel E Uy K Impact of Donors Aged 60 Years or More on Outcome after Lung Transplantation: Results of an 11-year Single-center Experience. J Thorac Cardiovasc Surg (2007) 133(2):525–31. 10.1016/j.jtcvs.2006.09.054 17258592

[11] Baldwin MR Peterson ER Easthausen I Quintanilla I Colago E Sonett JR Donor Age and Early Graft Failure after Lung Transplantation: a Cohort Study. Am J Transpl (2013) 13(10):2685–95. 10.1111/ajt.12428 PMC4157369 24034167

[12] Snell GI Yusen RD Weill D Strueber M Garrity E Reed A Report of the ISHLT Working Group on Primary Lung Graft Dysfunction, Part I: Definition and Grading-A 2016 Consensus Group Statement of the International Society for Heart and Lung Transplantation. J Heart Lung Transpl (2017) 36(10):1097–103. 10.1016/j.healun.2017.07.021 28942784

[13] Verleden GM Glanville AR Lease ED Fisher AJ Calabrese F Corris PA Chronic Lung Allograft Dysfunction: Definition, Diagnostic Criteria, and Approaches to Treatment-A Consensus Report from the Pulmonary Council of the ISHLT. J Heart Lung Transpl (2019) 38(5):493–503. 10.1016/j.healun.2019.03.009 30962148

[14] Ius F Aburahma K Boethig D Salman J Sommer W Draeger H Long-term Outcomes after Intraoperative Extracorporeal Membrane Oxygenation during Lung Transplantation. J Heart Lung Transpl (2020) 39(9):915–25. 10.1016/j.healun.2020.04.020 32444157

[15] Tudorache I Sommer W Kühn C Wiesner O Hadem J Fuhner T Lung Transplantation for Severe Pulmonary Hypertension–Awake Extracorporeal Membrane Oxygenation for Postoperative Left Ventricular Remodelling. Transplantation (2015) 99(2):451–8. 10.1097/TP.0000000000000348 25119128

[16] Smits JM van der Bij W Van Raemdonck D de Vries E Rahmel A Laufer G Defining an Extended Criteria Donor Lung: an Empirical Approach Based on the Eurotransplant Experience. Transpl Int (2011) 24(4):393–400. 10.1111/j.1432-2277.2010.01207.x 21155901

[17] Sommer W Kirschner H Ius F Salman J Siemeni T Bobylev D Transplantation of Donor Lungs with Pulmonary Embolism - a Retrospective Study. Transpl Int (2019) 32(6):658–67. 10.1111/tri.13407 30712278

[18] Terada Y Gauthier JM Pasque MK Takahashi T Liu J Nava RG Clinical Outcomes of Lung Transplants from Donors with Unexpected Pulmonary Embolism. Ann Thorac Surg (2021) 112(2):387–94. 10.1016/j.athoracsur.2020.08.040 33506764 PMC8060353

[19] Halpern SE Jawitz OK Raman V Choi AY Haney JC Klapper JA Aggressive Pursuit and Utilization of Non-ideal Donor Lungs Does Not Compromise post-lung Transplant Survival. Clin Transpl (2021) 35(9):e14414. 10.1111/ctr.14414 PMC8556224 34218467

[20] Urlik M Latos M Antończyk R Nęcki M Kaczur E Miernik M Suboptimal Donors Do Not Mean Worse Results: A Single-Center Study of Extending Donor Criteria for Lung Transplant. Transpl Proc. (2020) 52(7):2123–7. 10.1016/j.transproceed.2020.03.042 32482452

[21] Schwarz S Rahimi N Kifjak D Frommlet F Benazzo A Jaksch P Lungs from Polytrauma Donors with Significant Chest Trauma Can Be Safely Used for Transplantation. J Thorac Cardiovasc Surg (2022) 163(5):1719–31.e2. 10.1016/j.jtcvs.2020.10.150 33451825

[22] Fischer S Gohrbandt B Struckmeier P Niedermeyer J Simon A Hagl C Lung Transplantation with Lungs from Donors Fifty Years of Age and Older. J Thorac Cardiovasc Surg (2005) 129(4):919–25. 10.1016/j.jtcvs.2004.07.053 15821664

[23] Dahlman S Jeppsson A Scherstén H Nilsson F. Expanding the Donor Pool: Lung Transplantation with Donors 55 Years and Older. Transpl Proc. (2006) 38(8):2691–3. 10.1016/j.transproceed.2006.07.037 17098041

[24] Chambers DC Cherikh WS Harhay MO Hayes D Jr Hsich E Khush KK The International Thoracic Organ Transplant Registry of the International Society for Heart and Lung Transplantation: Thirty-Sixth Adult Lung and Heart-Lung Transplantation Report-2019; Focus Theme: Donor and Recipient Size Match. J Heart Lung Transpl (2019) 38(10):1042–55. 10.1016/j.healun.2019.08.001 PMC6816340 31548030

[25] Mulligan MJ Sanchez PG Evans CF Wang Y Kon ZN Rajagopal K The Use of Extended Criteria Donors Decreases One-Year Survival in High-Risk Lung Recipients: A Review of the United Network of Organ Sharing Database. J Thorac Cardiovasc Surg (2016) 152(3):891–8.e2. 10.1016/j.jtcvs.2016.03.096 27234027

[26] Scarborough JE Bennett KM Davis RD Lin SS Tracy ET Kuo PC Temporal Trends in Lung Transplant center Volume and Outcomes in the United States. Transplantation (2010) 89(6):639–43. 10.1097/TP.0b013e3181ceecf7 20075790

[27] Yang Z Subramanian MP Yan Y Meyers BF Kozower BD Patterson GA The Impact of Center Volume on Outcomes in Lung Transplantation. Ann Thorac Surg (2022) 113(3):911–7. 10.1016/j.athoracsur.2021.03.092 33857492 PMC8505551

[28] Desai R Collett D Watson CJ Johnson P Evans T Neuberger J. Cancer Transmission from Organ Donors-Unavoidable but Low Risk. Transplantation (2012) 94(12):1200–7. 10.1097/TP.0b013e318272df41 23269448

[29] Desai R Collett D Watson CJ Johnson P Evans T Neuberger J. Estimated Risk of Cancer Transmission from Organ Donor to Graft Recipient in a National Transplantation Registry. Br J Surg (2014) 101(7):768–74. 10.1002/bjs.9460 24771410

[30] Greenhall GHB Ibrahim M Dutta U Doree C Brunskill SJ Johnson RJ Donor-Transmitted Cancer in Orthotopic Solid Organ Transplant Recipients: A Systematic Review. Transpl Int (2022) 35:10092. 10.3389/ti.2021.10092 35185366 PMC8842379

[31] Benissan-Messan DZ Hayanga AJ Hayanga HE Morrell M Huffman L Shigemura N Contemporary Analysis of Early Outcomes after Lung Transplantation in the Elderly Using a National Registry. J Heart Lung Transpl (2015) 34(2):182–8. 10.1016/j.healun.2014.09.028 25447584

[32] Eurotransplant. Wp Content Uploads 2020 01 H4 Kidney 2021.2 April 2021 (2022). Available at: https:www.eurotransplant.org/wp-content/uploads/2020/01/H4-Kidney-2021.2-April-2021.pdf (Accessed May 3, 2022).

[33] OPTN. National Data (2022). Available at: https://optn.transplant.hrsa.gov/data/view-data-reports/national-data/; https://www.eurotransplant.org/wp-content/uploads/2022/03/ET_AR2020_LR_def.pdf (Accessed May 3, 2022).

[34] Hall DJ Jeng EI Gregg JA Pelaez A Emtiazjoo AM Chandrashekaran S The Impact of Donor and Recipient Age: Older Lung Transplant Recipients Do Not Require Younger Lungs. Ann Thorac Surg (2019) 107(3):868–76. 10.1016/j.athoracsur.2018.09.066 30444994

[35] Hayanga AJ Aboagye JK Hayanga HE Morrell M Huffman L Shigemura N Contemporary Analysis of Early Outcomes after Lung Transplantation in the Elderly Using a National Registry. J Heart Lung Transpl (2015) 34(2):182–8. 10.1016/j.healun.2014.09.028 25447584

